# Comparative Volumetric Analysis of Hermes and Synapse Software Systems in the Setting of Liver Surgery

**DOI:** 10.1007/s11605-022-05407-9

**Published:** 2022-07-13

**Authors:** K. Joshi, A. Nutu, M. Wilson, R. Marudanayagam, J. Isaac, R. P. Sutcliffe, B. V. M. Dasari

**Affiliations:** 1 The HPB and Liver Transplant Unit, Queen Elizabeth Hospital, Edgbaston, Birmingham, B15 2WB UK; 2 Department of Nuclear Medicine, Queen Elizabeth Hospital, Birmingham, UK

Liver volumetry is routinely performed to calculate the total and remnant liver volumes to assess the risk of PHLF. Semi-automatic software programs such as Mevis, Synapse, Image J, and Osirix are commonly used to assess the liver volumes in a pre-operative setting.^1^ Hermes remnant liver analysis software (Medical Software Affinity viewer™, Hermes Medical Solutions, Inc, Sweden) is a newer platform mainly used in SPECT-CT studies. Its application has been extended to dynamic planar and SPECT/CT examination of the uptake and excretion of technetium^99^ mebrofenin allowing assessment of anatomical as well as functional liver volumes. Synapse Vincent® medical imaging system (Fujifilm Medical Co., Ltd., Tokyo, Japan), that is in use for liver volumetric studies, has the additional advantage of 3D rendering and automatic liver/vessel extraction for a better understanding of the relationship between tumour and vessels. The objective of this study is to evaluate the reliability of anatomical volumetric assessment performed using Synapse Vincent® and Hermes Medical Software Affinity viewer™ softwares in the setting of liver resection surgery. A retrospective liver volumetric analysis was performed using both the softwares on 144 patients who underwent right and left hemi-hepatectomy between January 2015 and March 2019 at Queen Elizabeth Hospital, Birmingham, UK. Twenty-eight out of 144 patients developed PHLF (19.4%)—grade A (13.0%), grade B (4.2%), and grade C (1.4%) (ISGLS definition). The dry weights of the specimens were obtained from the histopathology report. The median weight of the resected specimen was 823 g (IQR: 616.5–1080 g). The median estimated volume of the resected specimen with Synapse was 882.20 ml (IQR: 638–1104 ml), and with Hermes, it was 979.7 ml (IQR: 784.31 ml). Estimated FLR volume calculated from Synapse and Hermes showed a median value of 793 ml (IQR: 589–1059 ml) and 517 ml (IQR: 393.74), respectively.

In the literature, clinically significant over- or underestimation of the LV_Rem_% (by ≥ 5%) was reported in 31.9% patients undergoing major liver resection for colorectal liver metastases.^2^ There is also a difference between in vivo CT volumetry and ex vivo water displacement volumetry, which is likely due to blood perfusion of the liver.^3^ Body mass index is another factor to influence the difference between measured and estimated liver volumes (*P* < 0.001). Taking these factors into consideration, standardised liver volumes that account for a variety of different parameters (BSA, age, gender, weight, or height) were proposed.^4–6^

In the current study, there was a positive correlation between the volumes estimated by Synapse with that of Hermes volumetry (*R*^2^ = 0.71; *P* < 0.001). There was a positive correlation between actual weight of the resected specimen and estimated volumes of resection with Hermes (*R*^2^ = 0.70, *P* < 0.001) (Fig. 1) and Synapse (*R*^2^ = 0.89, *P* < 0.001) (Fig. 2). Significant positive correlation was demonstrated between sFLRs calculated by Hermes and Synapse softwares and sFLRs based on the Urata, Heineman, and Vauthey formulas with *R*^2^ values of 0.7 and 0.6, respectively (*P* value < 0.001). However, AUROC analyses of sFLRs in predicting PHLF by Hermes were better (AUC: 0.76; 95%CI: 0.659–0.864; *P*-value < 0.001) than that of Synapse (AUC: 0.68; 95%CI: 0.545–0.815; *P*-value 0.01). The study reports that both of these software systems provide comparable anatomical volumetric analyses that can be used in liver surgical practice.

**Fig. 1:**
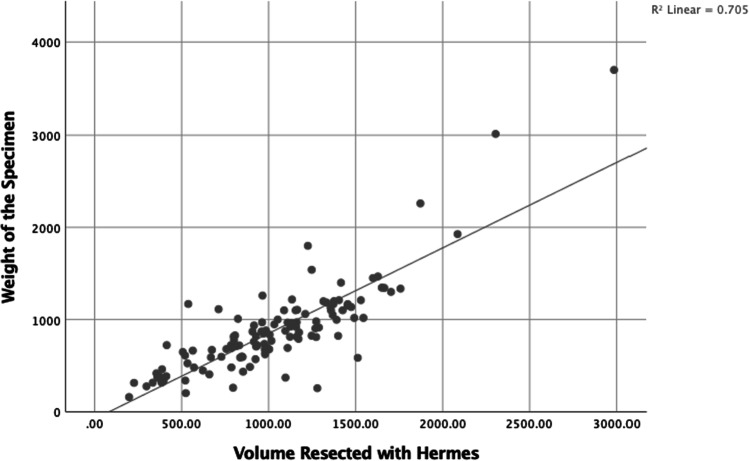
Scatter plot showing distribution between volumes resected with Hermes and actual weight of the specimen

**Fig. 2:**
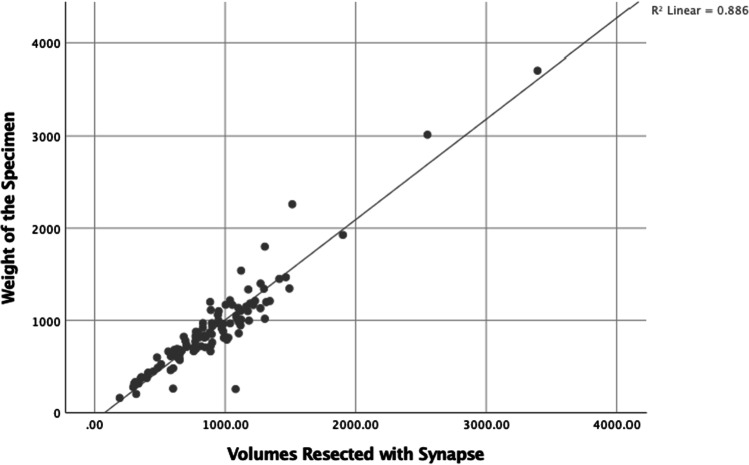
Scatter plot showing distribution between volumes resected with Synapse and actual weight of the specimen

## Abbreviations

FLR: Future liver remnant
PHLF: Post-hepatectomy liver failure
SPECT: Single photon emission computed tomography
sFLR: Standardised future liver remnant
SLV: Standardised liver volume
LV_Rem_%: Anatomical remnant volume
